# Impact of pressure recovery on the assessment of pulmonary homograft function using Doppler ultrasound

**DOI:** 10.14814/phy2.15432

**Published:** 2022-12-13

**Authors:** Jan‐Christian Reil, Christoph Marquetand, Claudia Busch‐Tilge, Jule Rohde, Edda Bahlmann, Anas Aboud, Ingo Eitel, Stephan Ensminger, Efstratios I. Charitos, Gert‐Hinrich Reil, Ulrich Stierle

**Affiliations:** ^1^ Medizinische Klinik II, Kardiologie, Angiologie und Internistische Intensivmedizin Universitäres Herzzentrum Lübeck, Universitätsklinikum Schleswig‐Holstein Lübeck Germany; ^2^ Klinik für Herzchirurgie Universitäres Herzzentrum Lübeck, Universitätsklinikum Schleswig‐Holstein Lübeck Germany; ^3^ Department of Cardiology Asklepios Kliniken St. Georg Hamburg Germany; ^4^ Department of Cardiac Surgery Kerckhoff Heart Center Bad Nauheim Germany; ^5^ Universitätsklinik für Innere Medizin – Kardiologie, Klinikum Oldenburg Oldenburg Germany

**Keywords:** energy loss index, homograft, pressure recovery, right ventricular afterload, Ross procedure, valvular hemodynamics

## Abstract

Relevant pressure recovery (PR) has been shown to increase functional stenotic aortic valve orifice area and reduce left ventricular load. However, little is known about the relevance of PR in the pulmonary artery. The study examined the impact of PR using 2D‐echocardiography in the pulmonary artery distal to the degenerated homograft in patients after Ross surgery. Ninety‐two patients with pulmonary homograft were investigated by Doppler echocardiography (mean time interval after surgery 31 ± 26 months). PR was measured as a function of pulmonary artery diameter determined by computed tomography angiography. Homograft orifice area, valve resistance, and transvalvular stroke work were calculated with and without considering PR. PR decreased as the pulmonary artery diameter increased (*r* = −0.69, *p* < 0.001). Mean PR was 41.5 ± 7.1% of the Doppler‐derived pressure gradient (*P*_max_), which resulted in a markedly increased homograft orifice area (energy loss coefficient index [ELCOI] vs. effective orifice area index [EOAI], 1.3 ± 0.4 cm^2^/m^2^ vs. 0.9 ± 0.4 cm^2^/m^2^, *p* < 0.001). PR significantly reduced homograft resistance and transvalvular stroke work (822 ± 433 vs. 349 ± 220 mmHg × ml, *p* < 0.0001). When PR was considered, the correlations of the parameters used were significantly better, and 11 of 18 patients (61%) in the group with severe homograft stenosis (EOAI <0.6 cm^2^/m^2^) could be reclassified as moderate stenosis. Our results showed that the Doppler measurements overestimated the degree of homograft stenosis and thus the right ventricular load, when PR was neglected in the pulmonary artery. Therefore, Doppler measurements that ignore PR can misclassify homograft stenosis and may lead to premature surgery.

## Graphical abstract

The presented results showed that the Doppler measurements overestimated the degree of homograft stenosis and thus the right ventricular load when the energy loss concept with pressure recovery (PR) in the pulmonary artery was neglected. Doppler measurements that ignore PR can misclassify the severity of homograft stenosis and may lead to premature surgery.

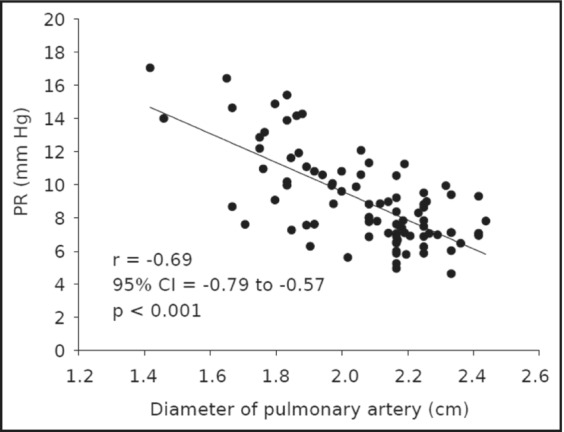

## 1 INTRODUCTION

Pressure recovery (PR) in aortic valve stenosis is an established physiological principle and was demonstrated experimentally (Heinrich et al., 1996) and clinically (Laskey & Kussmaul, 1994) using invasive pressure measurements. PR downstream from aortic valve stenosis defines the increase in pressure caused by reconversion of kinetic energy into potential energy in the aortic root (Baumgartner et al., 1999; Niederberger et al., 1996). The pressure gradient between the left ventricular outflow tract (LVOT) and the aortic root including PR is called energy loss (EL) and results in an increased functional orifice area (energy loss coefficient [ELCO]) when compared to the effective orifice area (EOA) calculated by Doppler echocardiography using the continuity equation (Akins et al., 2008; Baumgartner et al., 1999; Garcia et al., 2003). To overcome these limitations, equations using Doppler‐derived parameters were developed to calculate PR and ELCO considering the ascending aortic cross‐sectional area (CSA) (Baumgartner et al., 1999). The prognostic significance of the PR corrected parameters was previously demonstrated in patients with aortic valve stenosis (Altes et al., 2020; Bahlmann et al., 2013).

In contrast to the data on the left heart, less is known about the PR in the pulmonary artery distal to the pulmonary valve. In a simultaneous Doppler echocardiography and cardiac catheter correlative study, Singh et al. (2020) reported relevant PR in congenital pulmonary valve stenosis resulting in misclassification of the severity of Doppler‐derived stenotic valve lesion in 24 pediatric patients. In addition, the authors have presented convincingly that PR accounts for the discrepancy between Doppler *P*_max_ and catheter peak‐to‐peak gradient in aortic stenosis, coarctation of the aorta, and pulmonary stenosis and thus proved the concept of fluid mechanics in different vascular regions. The importance of PR in the pulmonary artery in patients with Ross procedure replacing the patient's diseased aortic valve by its own healthy pulmonary valve and substituting a homograft in the vacant pulmonary valve position, is unknown. The risk per year for degeneration of the homograft with relevant stenosis or regurgitation in the postoperative course is about 0.62% (Aboud et al., 2021). The aim of the study was to evaluate the occurrence and magnitude of PR in the pulmonary system in 92 patients of the European Ross Registry with relevant homograft stenosis after Ross surgery.

## 2 METHODS

### 2.1 Theoretical background for PR and EL

**FIGURE 1:**
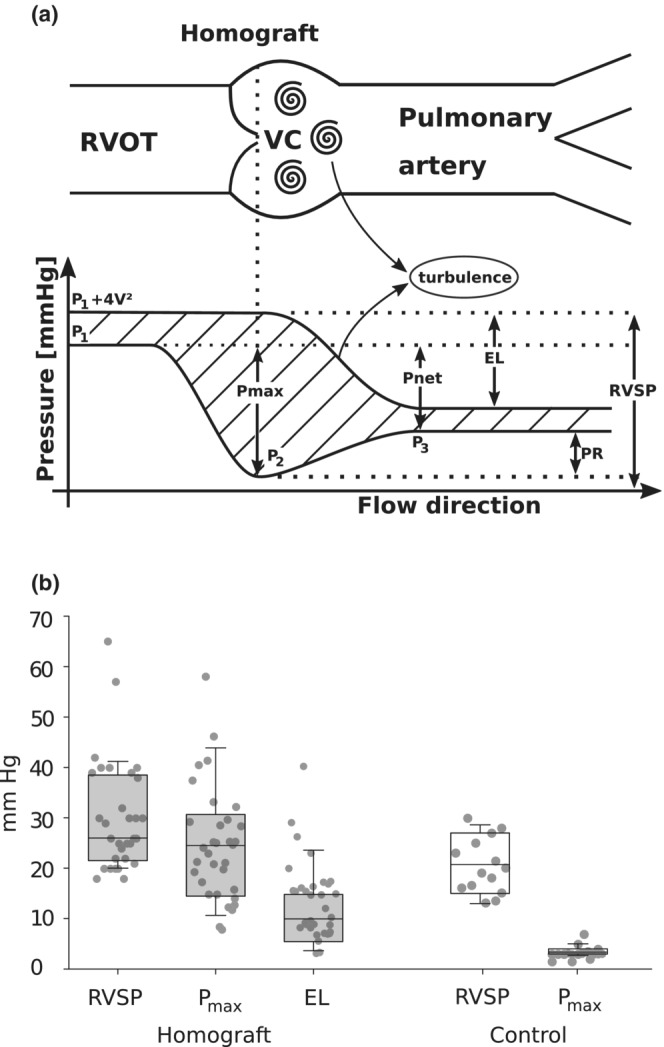
(a) Scheme showing the changes in pressure and energy when blood flows through the obstructed homograft into the pulmonary artery. For details, see Section 2 with definition of parameters. (b) Comparison of RVSP, *P*_max_, and EL in homograft patients with RVSP and *P*_max_ in controls. EL, energy loss; RVSP, right ventricular peak systolic pressure.

According to the basic concepts of hydraulics, there is a conversion of kinetic energy (4*V* ^2^) into heat in the post‐stenotic expansion section (Akins et al., 2008). Figure 1a summarizes the pressure and energy changes as blood flows from the right ventricular outflow tract (RVOT) through the obstructed homograft into the pulmonary artery in analogy to the left heart (Akins et al., 2008; Garcia et al., 2000). Distal to the vena contracta (*P*_max_ = *P*1 – *P*2) blood flow slows down and turbulence leads to EL by converting kinetic energy into heat (expansion section) (Akins et al., 2008). In addition, a smaller part of the kinetic energy is reconverted into potential energy (*P*3), which explains the PR phenomenon in the pulmonary artery root. The sum of kinetic and potential energy is therefore significantly reduced in the pulmonary artery compared to the RVOT and vena contracta. This difference is caused by the EL. When considering the potential energy alone (*P*1), the pressure gradient between RVOT and pulmonary artery is referred to as net pressure gradient (*P*_net_ = *P*1 − *P*3) (Akins et al., 2008).

### 2.2 Definition of parameters

$$\text{Maximal pressure gradient}:P_{\max}=P1-P2=4V_{\max}^{2}$$

*P*_max_ can be derived from Doppler measurements, where *V*_max_ is the maximum velocity derived from CW Doppler (Garcia et al., 2000).

$$\text{Energy loss}:\mathrm{EL}=4V_{\max}^{2}\times {\left(1-\mathrm{EOA}/P_{A}\right)}^{2}$$

where EOA is the effective orifice area of the homograft when using continuity equation and *P*_A_ is the CSA of the pulmonary artery. It is calculated by *P*_A_ = (*D*/2)^2^ × *π* (*D* = diameter of proximal pulmonary artery) (Baumgartner et al., 1999).

$$\begin{array}{c} \text{Pressure recovery}:\mathrm{PR}=P3-P2=4V_{\max}^{2}\times 2\mathrm{EOA}/P_{A}\\ \;\times \left(1-\mathrm{EOA}/P_{A}\right) \end{array}$$

Pressure recovery index (PRI):

$$\mathrm{PRI}=\mathrm{PR}/P_{\max}=P_{\max}\times 2\left(\mathrm{EOA}/P_{A}-{\left(\mathrm{EOA}/\mathrm{PA}\right)}^{2}\right)/P_{\max}$$

$$=\mathrm{PR}/P_{\max}=2\left(\mathrm{EOA}/P_{A}-{\left(\mathrm{EOA}/P_{A}\right)}^{2}\right)$$

$$=\mathrm{PR}/P_{\max}=C$$

According to Voelker et al. (1992) *C* can be calculated by the ratio of EOA and *P*_A_:

$$C=2\left(\mathrm{EOA}/P_{A}-{\left(\mathrm{EOA}/P_{A}\right)}^{2}\right)$$

The formula developed by Voelker et al. clearly shows that the relative pressure recovery (PRI = *C*) depends only on the ratio of valve opening area (EOA) and vessel CSA or in this case, the pulmonary CSA (EOA/*P*_A_) and mathematically describes the relationship between the two variables as an inverted parabola. The summit of PRI of 50% can be attained by EOA/*P*_A_ of 0.5. Smaller and higher ratios of EOA/*P*_A_ result in lower values of PRI indicated by the parabolic function. The EOA/*P*_A_ ratio covers the entire range of all possible degrees of valve stenosis and vessel CSAs. Interestingly, other factors such as flow and turbulence have no effect on this function. Absolute PR is also influenced by *P*_max_ (Baumgartner et al., 1999).

$$\mathrm{Net}\;\text{pressure}:P_{\mathrm{net}}=P1-P3=P_{\max}-\mathrm{PR}$$

Effective orifice area of homograft when using the continuity equation indexed to body surface area (BSA): EOAI.

Energy loss coefficient index (= functional opening area of homograft taking PR into account) (Garcia et al., 2000):

$$\text{ELCOI}=\mathrm{EOA}\times P_{A}/\left(P_{A}-\mathrm{EOA}\right)/\mathrm{BSA}$$

$$\text{Valve resistance}:\mathrm{Vr}=P_{\text{mean}}/Q\;\left(Q=\text{flow}\;\left[\mathrm{ml}/s\right]\right)$$

Valve resistance considering PR (Garcia et al., 2000):

$${\mathrm{Vr}}_{\mathrm{PR}}=\text{mean}\;\mathrm{EL}/Q$$

$$\text{mean}\;\mathrm{EL}=4V_{\text{mean}}^{2}\times {\left(1-\mathrm{EOA}/P_{A}\right)}^{2}$$

Transvalvular stroke work:

$$\mathrm{SW}=P_{\text{mean}}\times \mathrm{SV}\;\left(\mathrm{SV}=\text{stroke volume}\right)$$

Transvalvular stroke work considering PR:

$${\mathrm{SW}}_{\mathrm{PR}}=\text{mean}\;\mathrm{EL}\times \mathrm{SV}$$

### 2.3 Patients

Ninety‐two patients with the Ross procedure (mean age of 42 ± 12.7 years; 14 females, 78 males) were routinely investigated by echocardiography between 2012 and 2018 as part of the Ross registry follow‐up protocol (Aboud et al., 2021). Participation took place on a voluntary basis. Patients showed sinus rhythm, a normal LV ejection fraction (EF: 60 ± 10%), and no other severe valvular heart disease. In these patients, computed tomography (CT) angiography was additionally performed due to a newly developed pressure gradient across the homograft, suspected calcification, or fever of unknown etiology. The mean time interval between the initial surgery and CT angiography was 29 ± 35 months (range: 5–156 months). At this time, the maximum homograft gradient was 19.7 ± 9.6 mmHg and the mean 9.8 ± 6.5 mmHg (Table 2). Five patients had grade I homograft regurgitation and two patients had grade II regurgitation. All patients provided their informed consent for the collection and use of the respective data. The study was approved by the local ethics committee (No. 20‐399) and is a subanalysis of the European Ross Registry (Clinical Trials ID: NCT 00708409).

### 2.4 Echocardiographic measurements

Transthoracic Doppler echocardiography was carried out with a Vivid 9 system (GE Healthcare). Details of echocardiographic evaluation have been previously described (Reil et al., 2020). The investigations and analyses of the acquired echocardiographic data were performed by two investigators.

### 2.5 Valvular hemodynamics

Mean and maximal transvalvular pressure gradient (*P*_mean_/*P*_max_) were assessed by using the CW‐Doppler signal across the pulmonary homograft. Stroke volume was assessed using the diameter of the RVOT as well as pulsed wave Doppler VTI of RVOT. SV = *D* ^2^/4 × *π* × VTI_RVOT_ where *D* is the diameter of RVOT. The dimensionless velocity index (DVI) was calculated by the ratio of VTI_RVOT_ and transvalvular VTI collected by CW‐Doppler. The diameter of the pulmonary artery was measured 2 cm beyond the mobile parts of the homograft using parasternal short axis. The ventricular ejection time (*t*) was measured by assessing the time between the R‐wave of ECG and the end of the pulsed‐waved Doppler RVOT‐VTI signal (Zoghbi et al., 2017). Flow was calculated by *Q* = SV/*t*.

In a homograft subgroup of 33 patients, the right ventricular peak systolic pressure (RVSP) could also be measured using the tricuspidal regurgitation Doppler signal as a reliable approximation of total right ventricular afterload. Additionally, in 26 healthy patients with native pulmonary valves, RVSP and *P*_max_ were assessed.

### 2.6 CT angiography examination

All CT angiographies were performed during apnea, using identical settings (Toshiba Medical Systems, Aquilion Multi; length of scan 12 cm, 2 mm slices, 0.5 s rotation time, 120 kV, 150 mA) (Charitos et al., 2011). A CT scan either without contrast agent (to localize and quantify any calcified lesions) or with contrast agent (to evaluate the RVOT and pulmonary artery lumen morphology) was performed in all patients. In each patient, 120 ml of contrast agent (Ultravist 370®; Bayer HealthCare Pharmaceuticals) was injected via a peripheral vein at an infusion rate of 3.5 ml/s. In order to evaluate the examinations, electrocardiographic‐gated CT reconstructions were performed. Multiplane reconstructions in the paraaxial, parasagittal, and paracoronary planes were carried out to evaluate the RVOT and the main pulmonary artery, up to the level of the bifurcation. The pulmonary artery diameter was measured about 2 cm above the mobile portion of the homograft and above its distal suture in the paracoronary projection. A circular shape was assumed to calculate the vessel area.

### 2.7 Statistics

Continuous variables are presented as mean ± standard deviation. Before starting the statistical analysis, a Kolmogorov–Smirnov test for checking normal distribution of the samples was performed. Changes in hemodynamic parameters were analyzed and compared using two‐tailed Student's *t*‐test, if the data were normally distributed. Otherwise, a Mann–Whitney *U*‐test was used. The correlation coefficient of the Pearson product moment was calculated for variables with a linear relationship, whereas the Spearman correlation coefficient (rho; see Figure 4a–d) was calculated for variables with a monotonic but clearly nonlinear relationship. A comparison of correlation coefficients was performed using the cocor framework and R package (Diedenhofen & Musch, 2015). To compare the Pearson product‐moment correlation coefficients between two pairs of variables using the cocor framework (Diedenhofen & Musch, 2015), the inverse transformation was used to restore linearity before the Pearson product‐moment correlation coefficient was calculated and compared. *p*‐Values <0.05 were considered to reflect statistically significant differences. Statistical analyses were performed with SigmaPlot 14.5, Düsseldorf, Germany, and R version 4.1.1 (R Core Team, 2021).

## 3 RESULTS

Table 1 summarizes the basic clinical, echocardiographic, and technical homograft data of the investigated patients (*n* = 92). Most of these patients were male and had normal left and right ventricular function with only a minority of patients showing the typical cardiovascular risk factors. The main Doppler‐hemodynamic parameters of the cohort with or without correction for PR are shown in Table 2.

**TABLE 1:** Basic clinical data of the investigated patients.

Age (years)	42.3 ± 12.7
Gender	F 14, M 78
BSA (m^2^)	1.99 ± 0.21
BMI (kg/m^2^)	24.5 ± 2.3
Diameter homograft (donor, mm)	22–24 mm *n* = 11
	25–27 mm *n* = 76
	28–30 mm *n* = 5
Echo parameters	
TAPSE (mm)	19 ± 3
RVEDD (mm)	38 ± 6
Pulmonary regurgitation I°	*n* = 5
Pulmonary regurgitation II°	*n* = 1
LV ejection fraction (%)	60 ± 10
LVEDD (mm)	56 ± 4
Comorbidities	
Diabetes mellitus	*n* = 2
Hypertension	*n* = 26
Renal failure	*n* = 9
CAD	*n* = 4
Smoker	*n* = 26
COPD	*n* = 0
Hyperlipidemia	*n* = 16
Surgical technique	
Homografts (pulmonary origin)	
RVOT suture line proximal	Continuous *n* = 92
RVOT suture line distal	Interrupted *n* = 5
	Continuous *n* = 69
	Combined *n* = 16
	Unknown *n* = 2

Abbreviations: BMI, body mass index; BSA, body surface area; CAD, coronary artery disease; COPD, chronic obstructive pulmonary disease; LVEDD, left ventricular end diastolic diameter; RVEDD, right ventricular end diastolic diameter; RVOT, right ventricular outflow tract; TAPSE, tricuspid annular plane excursion.

**TABLE 2:** Homograft hemodynamics with and without calculating pressure recovery and comparison of RVSP and *P*_max_ to a subgroup of healthy controls.

	Doppler	Doppler with PR	
*P*_max_/energy loss (mmHg)	19.7 ± 9.6	9.8 ± 6.2	*p* < 0.001
*P*_mean_/mean energy loss (mmHg)	9.2 ± 6.5	4.7 ± 3.6	*p* < 0.001
*P*_net_ (mmHg)		11.8 ± 6.2	
Pressure recovery (mmHg)		8.1 ± 4.0	
PRI × 100 (%)		41.5 ± 7.1	
EOA/*P*_A_	0.37 ± 0.16		
Transvalvular stroke work (mmHg × ml)	822 ± 433	349 ± 220	*p* < 0.0001
Resistance (dyne × s × cm^−5^)	66 ± 36	30 ± 23	*p* < 0.001
Pulmonary valve opening area (cm^2^/m^2^)	EOAI 0.9 ± 0.4	ELCOI 1.3 ± 0.4	*p* < 0.001

	CT	Echo	
*P*_A_ diameter (mm)	20.8 ± 4.2	19.0 ± 3.7	*p* < 0.007

Subgroups	Homografts (*n* = 33)	Controls (*n* = 26)	
RVSP (mmHg)	29.9 ± 10.9	21.2 ± 6.0	*p* < 0.05
*P*_max_ (mmHg)	25.0 ± 12.8	3.6 ± 1.0	*p* < 0.0001
RVSP‐*P*_max_ (mmHg)	4.7 ± 4.7	17.6 ± 6.2	*p* < 0.0001
RVSP‐EL (mmHg)	18.4 ± 7.8		*p* = 0.67^a^

Abbreviations: CT, computed tomography; EL, energy loss; ELCOI, energy loss coefficient index; EOAI, effective orifice area index; PR, pressure recovery; PRI, pressure recovery index; RVSP, right ventricular peak systolic pressure.

^a^ *p* = 0.67 indicates comparison of RVSP‐EL (homograft) versus RVSP‐*P*_max_ (control).

In the subgroup of 33 homograft patients and 26 controls, the Doppler RVSP could be measured, which reflects the total valvular and pulmonary vascular resistance (RV afterload). The difference between RVSP and Pmax indicates the calculated pure pulmonary vascular resistance in mmHg. The difference between RVSP and *P*_max_ was small (<5 mmHg) in the homograft subgroup, but large in healthy controls (>17 mmHg). The difference between RVSP and EL (defining the sum of potential and kinetic energy lost) was significantly higher and corresponds exactly to the difference of RVSP‐*P*_max_ of healthy controls as a reference point (18.4 ± 7.8 vs. 17.6 ± 6.2 mmHg, *p* = 0.67) (Figure 1b; Table 2).

**FIGURE 2:**
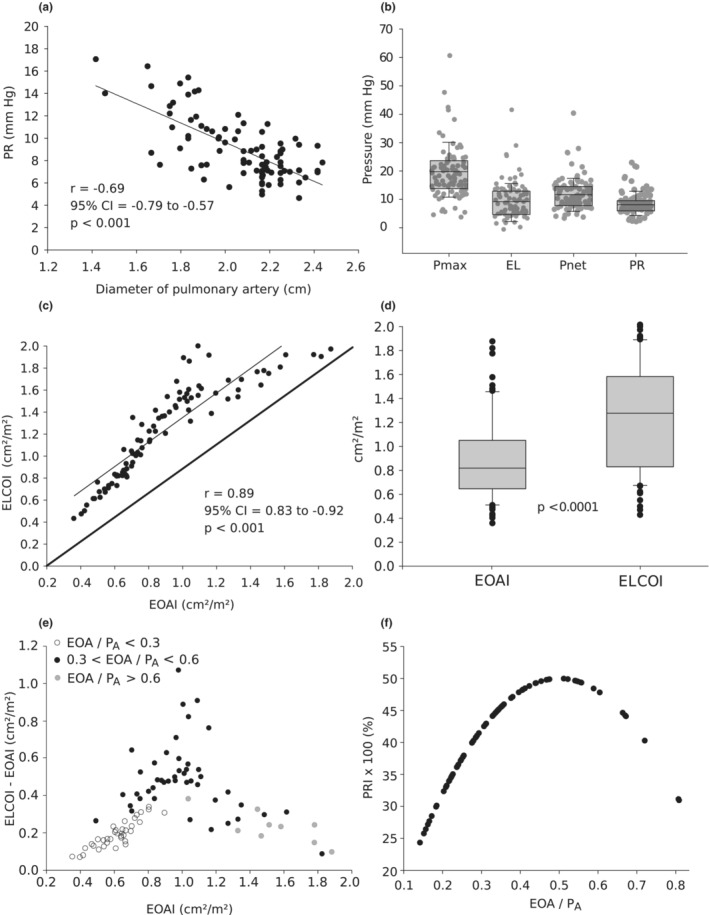
(a) Correlation between PR and the diameter of the pulmonary artery measured by CT scan. (b) Comparison of *P*_max_, energy loss, net pressure, and PR. (c) Correlation between ELCOI and EOAI. (d) Comparison of ELCOI and EOAI. (e) Correlation between the difference of ELCOI‐EOAI and EOAI. (f) Relation between PRI × 100 and the ratio of EOA/*P*_A_ for PRI = 2 (EOA/*P*_A_ − (EOA/*P*_A_)^2^). CT, computed tomography; ELCOI, energy loss coefficient index; EOAI, effective orifice area index; PR, pressure recovery; PRI, pressure recovery index.

A significant inverse correlation between PR and the diameter of pulmonary artery (CT scan) is depicted in Figure 2a. With increasing pulmonary artery diameter, PR decreases (*r* = −0.69, *p* < 0.001). Echo‐ and CT‐derived diameter measurements of the pulmonary artery showed good agreement (*r* = 0.84; 95% CI: 0.75–0.90; *p* < 0.001, Table 2). The relative mean proportion of PR to the Doppler Pmax was 41.5 ± 7.1% (Table 2). *P*_net_ and EL showed a low but significant difference (11.8 ± 6.2 vs. 9.8 ± 6.2 mmHg, *p* < 0.001) (Figure 2b, Table 2) and demonstrated a good correlation (*r* = 0.97; *r* ^2^ = 0.95; *p* < 0.0001, not shown). Both parameters are significantly reduced compared to *P*_max_ (*p* < 0.001).

ELCOI and EOAI showed a significant correlation with the correlation line shifted above the identity line (Figure 2c). The mean values of ELCOI and EOAI were 1.3 ± 0.4 and 0.9 ± 0.4 cm^2^/m^2^, respectively (Figure 2d) Additionally, the latter correlation shows an unequal distribution (heteroscedasticity), which becomes obvious when the difference between EOAI and ELCOI is plotted against EOAI (Figure 2e). The difference between ELCOI and EOAI depicts a maximum at an EOAI value around 1.0 and then decreases almost symmetrically toward both sides. The difference of both parameters was statistically significant (mean paired difference: 0.34 cm^2^/m^2^, 95% CI's: 0.30–0.38 cm^2^/m^2^, *p* < 0.001) and reveals an implied parabolic function. The parabolic curve of calculated PRI values (*C*, see Section 2) versus the ratio of the homograft orifice area (EOA) to the pulmonary artery CSA (*P*_A_) shows an optimum effect for the relative PR of 50% with a ratio of EOA to *P*_A_ of 0.5 (Figure 2f) (Voelker et al., 1992). The PRI and EOA/*P*_A_ values of the included patients are ideally distributed over the entire course of the curve and have a mean ratio of EOA/*P*_A_ of 0.37 ± 0.16. Absolute PR depends on Pmax and PRI resulting in a good correlation between Pmax and PR (*r* = 0.93, *p* < 0.001). The biggest difference between ELCOI and EOAI was noted in the EOAI range of 1.0 (Figure 2e), which is associated with an increased occurrence of EOA/*P*_A_ values between 0.3 and 0.6 (red colored points) and indicates the highest PRI results of the studied cohort. Higher grade (<0.3, blue colored points) and lower grade stenoses (>0.6 green colored points) show smaller differences.

**FIGURE 3:**
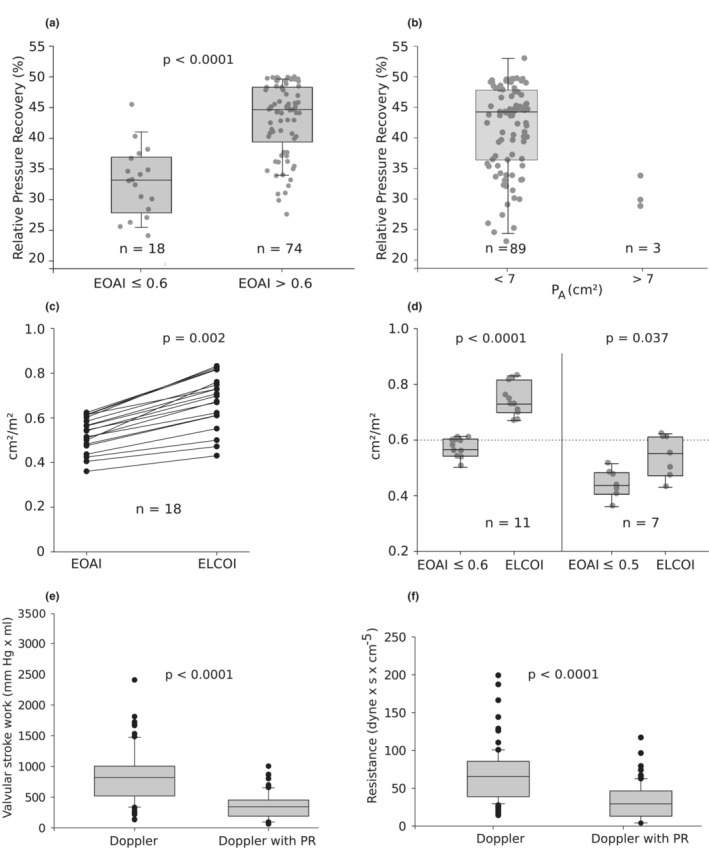
(a) Comparison of PRI (%) in patients with EOAI ≤0.6 cm^2^/m^2^ and >0.6 cm^2^/m^2^. (b) Comparison of PRI (%) in patients with *P*_A_ <7 cm^2^ and >7 cm^2^. (c) Comparison of EOAI and ELCOI in patients with severe homograft stenosis (EOAI ≤0.6 cm^2^/m^2^). (d) Comparison of EOAI and ELCOI in patients with severe homograft stenosis with EOAI ≤0.6 cm^2^/m^2^ and EOAI ≤0.5 cm^2^/m^2^. (e) Comparison of transvalvular stroke work calculated with and without considering PR. (f) Comparison of valvular resistance calculated with and without considering PR. ELCOI, energy loss coefficient index; EOAI, effective orifice area index; PR, pressure recovery; PRI, pressure recovery index.

A significantly smaller PRI (mean, 33 ± 0.5 vs. 43 ± 0.6%, *p* < 0.0001) was noted when the standardized homograft orifice area was ≤0.6 cm^2^/m^2^ (severe stenosis) compared to >0.6 cm^2^/m^2^ (Figure 3a), although overlap was shown in both groups. Similarly, a markedly smaller PRI (mean, 32 ± 0.5 vs. 42 ± 0.7%) was assessed when the *P*_A_ was >7 cm^2^ (= CS diameter >3 cm) than when it was <7 cm^2^ (Figure 3b). A direct comparison between EOAI <0.6 cm^2^/m^2^ and their corresponding ELCOI values in patients with severe homograft stenosis showed a significant difference (mean, 0.53 ± 0.08 vs. 0.68 ± 0.13 cm^2^/m^2^, *p* < 0.002), which indicates systematically higher ELCOI values (Figure 3c). After correction for the PR in this group of 18 patients, 11 of them (61%) could be reclassified as moderate stenoses (mean, EOAI 0.56 ± 0.04 vs. ELCOI 0.74 ± 0.06 cm^2^/m^2^, *p* < 0.0001). The seven patients with an EOAI value <0.5 cm^2^/m^2^ remained in the group of severe homograft stenoses even after PR correction (mean EOAI 0.44 ± 0.05 vs. ELCOI 0.54 ± 0.08 cm^2^/m^2^, *p* < 0.04) (Figure 3d).

**FIGURE 4:**
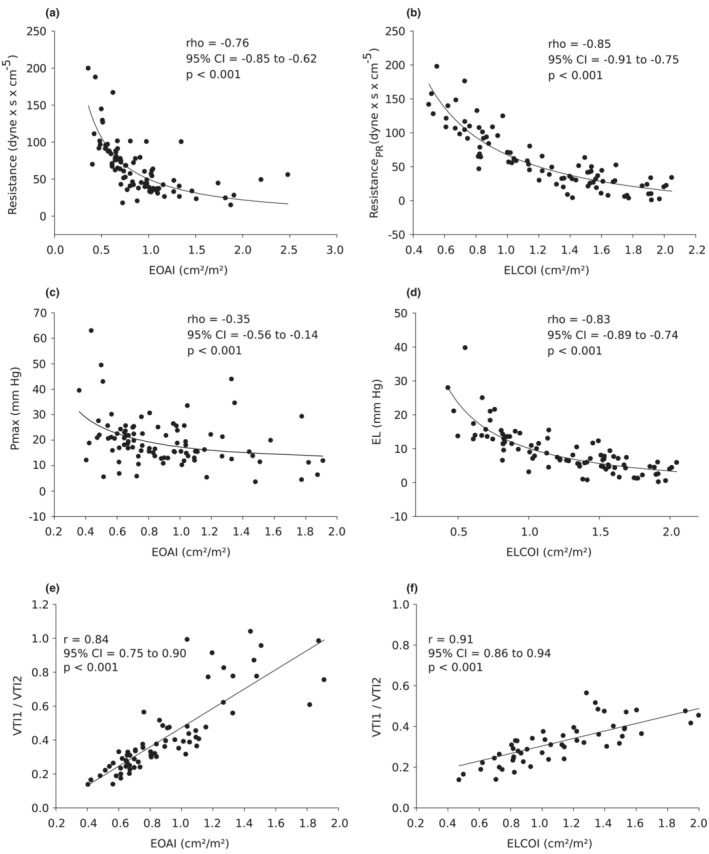
(a) Correlation of *P*_max_ and EOAI, (b) correlation of EL and ELCOI, (c) correlation of valve resistance (Vr) and EOAI, (d) correlation of valve resistance Vr_PR_ and ELCOI. Correlation of DVI (VTI1/VTI2) with EOAI (e) and ELCOI (f). DVI, dimensionless velocity index; ELCOI, energy loss coefficient index; EOAI, effective orifice area index.

Transvalvular stroke work and valvular resistance (Figure 3e,f; Table 2) are significantly lower (both >50%) if the Doppler measurements corrected for PR are taken into account, which thus show the true additional burden imposed on the right ventricle due to the homograft stenosis. The correlation of Vr_PR_ with 1/ELCOI (*r* = −0.86) was significantly better (*r*‐difference: 0.29; 95% CIs: 0.17–0.45; *p* < 0.001) compared to the correlation of Vr with 1/EOAI [*r* = −0.56 (Figure 4a,b)]. Identical results were obtained when EL was correlated with 1/ELCOI (*r* = −0.82) and was significantly better (*r*‐difference: 0.42; 95% CIs: 0.59–0.29; *p* < 0.001) than the correlation of *P*_max_ with 1/EOAI (*r* = −0.39) (Figure 4c,d).

The DVI can also be used as an additional parameter for grading the severity of a valve stenosis. Therefore, the correlation between DVI and EOAI (*r* = 0.84) as well as for ELCOI was tested. In this case, the correlation between DVI and ELCOI (*r* = 0.91) was significantly better as well (r‐difference: 0.08; 95% CIs: 0.02–0.15; *p* = 0.01) (Figure 4e,f). The calculation of the linear regression for the DVI and ELCOI resulted in the equation:

$$\mathrm{DVI}=0.12+\left(0.18\times \text{ELCOI}\right)$$

(Intercept 0.12 [95% CI: 0.08–016, *p* < 0.0001]; slope 0.18 [95% CI: 0.16–0.20, *p* < 0.001, *r* ^2^ = 0.83]). If the cutoff value for high‐grade stenoses of the ELCOI of 0.6 cm^2^/m^2^ is inserted in this equation, a DVI value of 0.23 (95% CI: 0.20–0.26) is calculated. This value roughly corresponds to a VTI ratio of  1 to 4, similar to that for severe aortic valve stenosis (Michelena et al., 2013).

## 4 DISCUSSION

### 4.1 Non‐invasive evidence of the EL concept in the pulmonary artery

Important clinical evidence for the relevance of the EL concept in the pulmonary artery can be derived non‐invasively from the RVSP measurements, which represent the calculated total afterload of the RV, and from the difference between Doppler RVSP and *P*_max_ in a subgroup of healthy controls and that between RVSP and EL in a subgroup of homograft patients. Both differences represent the calculated pure pulmonary vascular resistance and were almost the same, which was to be expected in both patient subgroups with healthy lungs. In contrast, without considering the EL concept, the difference between RVSP and *P*_max_ in the homograft patients was too small, because *P*_max_ was of the same order of magnitude as RVSP. In this calculation, the actual pulmonary vascular resistance was significantly underestimated compared to the correspondingly large difference in healthy controls as a reference point. This discrepancy was eliminated by applying the EL concept and hence justifies this approach in patients with stenosed homograft in the pulmonary vasculature. The RVSP is an extremely useful parameter in everyday clinical practice as it includes the PR distal to the vena contracta, and therefore indicates the true RV afterload, and can be used as “clinical control” for the use of the El concept.

### 4.2 Occurrence and magnitude of PR in the post‐stenotic pulmonary artery

Based on the EL concept, the PR is of higher magnitude if the vessel diameter downstream of a stenotic valve lesion is relatively small (Akins et al., 2008; Pibarot et al., 2013). This applies in particular to the pulmonary artery, which has an average diameter of less than 2.5 cm (Truong et al., 2012) compared to the aorta (approx. 3 cm) (Lang et al., 2015). Our results demonstrated a clear inverse correlation between the pulmonary artery diameter and PR showing that the EL principle is effective on the right heart in close agreement with the recent correlated study on simultaneous invasive and Doppler measurements in pediatric patients (Singh et al., 2020). ELCOI and EOAI showed a significant correlation with a general overestimation of the stenosis by the PR uncorrected EOAI. This overestimation was in particular true for mild and moderate stenosis, (range of hyperscedasticity) but was even detected in severe homograft stenosis (EOAI ≤0.6 cm^2^/m^2^) which resulted in a reclassification of 61% (11 out of 18 patients) from severe to moderate homograft stenosis. This result can be explained by the high mean PRI (33 ± 5%) found in this group, which is well above the clinically relevant PRI value of >20% (Bahlmann et al., 2010), and may therefore play an important role in the management of homograft patients in the transition area of severe to moderate homograft stenosis (0.5–0.6 cm^2^/m^2^).

The PR in relative terms (PRI) depends on the ratio of the valve orifice area and the CSA of the downstream vessel root. This relationship describes mathematically the interaction or “crosstalk” (El‐Hamamsy et al., 2021) between the ventricle, its obstructed valve, and adjacent artery by defining PRI in any vascular system that is subject to the law of hydraulics (Akins et al., 2008; Voelker et al., 1992). It is noteworthy that the mean PRI averages about 40% of the pulmonary Pmax in the homograft cohort, whereas a significantly lower relative PR (mean value of 16%) was reported in 1563 patients with aortic stenosis in the SEAS substudy (Bahlmann et al., 2010). The pulmonary artery is on average smaller than the aorta (Lang et al., 2015; Truong et al., 2012), thereby favoring a higher EOA/P_A_ ratio. Therefore, a higher extent of PR values may therefore be more common in the pulmonary artery than in the aorta. According to this, 89 of the 92 patients of our cohort showed a CSA of <7 cm^2^ which corresponds to the cutoff value of vessel diameter of <3 cm at which a clinically relevant PR can be expected (Baumgartner et al., 1999). In addition, our homograft cohort consists of a large proportion of patients with moderate homograft stenoses, who also favors a higher ratio of EOA/*P*_A_. Relative PR may be even more pronounced in Ross patients, as the implantation of the homograft conduit led to further shrinkage of the vessel (Charitos et al., 2011).

### 4.3 PR and unloading of the right ventricle

Loss of energy is a normal feature of the circulation when mechanical energy of the flowing blood is converted into heat by the friction of the vessel wall (Akins et al., 2008). However, in valvular stenosis, the ventricle needs additional energy to compensate for the higher ventricular load due to increased transvalvular stroke work. Therefore, reliable PR measurements are required to assess the actual ventricular load for clinical decision‐making. It is noteworthy that the calculated mean values of the RV transvalvular stroke work and valvular resistance dropped significantly to more than 50% as a result of the PR correction. This aspect is overlooked if PR is not calculated. Also, the correlations of Vr and *P*_mean_ were significantly better when the PR calculation was considered. It follows that PR seems to play a more effective role in unloading the RV than in the LV, since the pulmonary system pressure (<25 mmHg), the total resistance (approx. 180 dyne × s × cm^−5^), and the diameter of the pulmonary artery (<2.5 cm) are significantly lower than in the left system.

### 4.4 Clinical impact of DVI

The echocardiographic representation of the pulmonary valve can be challenging. Therefore, the optimal alignment of the Doppler beam and the stenotic jet is often a serious problem. The DVI can help in such cases. The DVI is another parameter for diagnosing high‐grade valve stenosis. A ratio of about <0.25 indicates high‐grade aortic stenosis regardless of Doppler direction but varies slightly with outflow tract diameter (Lang et al., 2015; Michelena et al., 2013). So far, there are only few comments available on this subject with respect to pulmonary valve (Haddy, 2021) but no substantiated data. The presented results showed a significant better correlation between ELCOI and DVI compared to EOAI (Figure 4e,f). Linear regression analysis provided a calculated DVI of 0.23 as a cutoff value for a severe homograft stenosis of ELCOI (<0.6 cm^2^/m^2^) is inserted in the equation (Bahlmann et al., 2013). This measurement is easy to handle, provides reliable results, and could therefore be of great practical importance, especially in the case of restricted echocardiographic conditions.

### 4.5 Limitations

The data presented here are all based on non‐invasive Doppler echocardiographic measurements, invasive measurements are missing. However, the equations used are all invasively validated on the left heart (Baumgartner et al., 1999; Garcia et al., 2000, 2003; Niederberger et al., 1996) and on the right heart in pediatric patients with pulmonary stenosis and other vasculature (Singh et al., 2020). Remarkably, clinical evidence for the EL concept in the pulmonary artery could be demonstrated non‐invasively using the Doppler technique, since the calculated pulmonary vascular resistance was only correctly reflected when the EL was taken into account. We are also unable to make any statements regarding the prognostic significance of the presented results. In the left heart, however, parameters such as the ELCOI are of great prognostic importance (Altes et al., 2020; Bahlmann et al., 2010; Bahlmann et al., 2013).

## 5 CONCLUSION

The presented results show that relevant PR with overestimation of the degree of pulmonary homograft stenosis occurred in patients with the Ross procedure when uncorrected Doppler measurements were performed. When PR was considered, a significant reduction in transvalvular resistance and RV stroke work was noted, and 61% of homograft patients with severe stenosis were reclassified to moderate stenosis.

Based on these data, it can be assumed that the degree of homograft stenosis was assessed as too high in the past, possibly leading to misclassification and premature interventions.

## AUTHOR CONTRIBUTIONS

**Jan‐Christian Reil:** Conception of the study, performing echoes, interpreting data, drafting the manuscript. **Christoph Marquetand:** Statistical analysis, drafting the figures, critically reviewing the final manuscript. **Jule Rohde:** Collection of data, critically reviewing the final manuscript. **Claudia Busch‐Tilge:** Critically reviewing the final manuscript. **Edda Bahlmann:** Critically reviewing the final manuscript. **Anas Aboud:** Critically reviewing the final manuscript, performing surgery (DY, Ross). **Ingo Eitel:** Critically reviewing the final manuscript. **Stephan Ensminger:** Critically reviewing the final manuscript. **Efstratios I. Charitos:** Statistical analysis, critically reviewing the final manuscript. **Gert‐Hinrich Reil:** Conception of the study, interpreting data, drafting the manuscript. **Ulrich Stierle:** Conception of the study, performing echoes, interpreting data, critically reviewing the final manuscript.

## ETHICS STATEMENT

Written informed consent of all subjects participating in the research comprising the manuscript was obtained. The procedures were in accordance with the ethical standards of the responsible committee on human experimentation (institutional and national) and with the Helsinki Declaration of 1975, as revised in 2008.

## CONFLICTS OF INTEREST

All authors declared no conflict of interest.
